# Doctors' Fees and Nursing Homes

**Published:** 1912-09-07

**Authors:** 


### DOCTORS' FEES AND NURSING HOMES.

Our previous leading article on this subject has had the effect of drawing further information from "Fair Game," whose grievances described in the *Times* were the subject of discussion. These grievances he now details at some length and with some humour, but his charge is a grave one. His doctor, he says, lured him into a nursing home on insufficient grounds, and there made him, together with the other patients, subject to a routine of visits which occurred invariably at the same times and days each week and whose net result is alleged to be the transference of guineas from the pockets of the patients to those of their medical attendant. Every profession has its scoundrels, no doubt, but the fact that "Fair Game" has been victimised, as seems probable from the further facts about his complaint which he gives in our correspondence columns, is not so interesting as his account of the way in which his own faith in his doctor was abused.

A considerable time elapsed before "Fair Game's" faith wilted away sufficiently to make him take any action. He lacked the courage to act on his fears: his knees knocked before the terms in the Medical Dictionary. He felt, in short, too much at a disadvantage. That, of course, is the case with the majority of patients, and it is difficult to know whether the profession or the public is the more to blame for it. The public demands "cures," of the working, principles, mode of which it knows and desires to know nothing. It shares the vulgar preference for miracles. The profession too, no doubt, has collectively been encouraged by this to appeal to the eager credulity of the public with the exaggeration of mystery and the pomp of strange words. Sometimes, as in the present case, a member of the public is startled by the suspicion that his credulity has been exploited. But, though the exploiter certainly deserves punishment, can the vast majority of patients, who take positive pride in their credulous ignorance, be wholly exonerated?

